# Geometric Calibration for Cameras with Inconsistent Imaging Capabilities

**DOI:** 10.3390/s22072739

**Published:** 2022-04-02

**Authors:** Ke Wang, Chuhao Liu, Shaojie Shen

**Affiliations:** Department of Electronic and Computer Engineering, Hong Kong University of Science and Technology, Hong Kong SAR, China; cliuci@connect.ust.hk (C.L.); eeshaojie@ust.hk (S.S.)

**Keywords:** geometric calibration, camera calibration, inconsistent imaging

## Abstract

Traditional calibration methods rely on the accurate localization of the chessboard points in images and their maximum likelihood estimation (MLE)-based optimization models implicitly require all detected points to have an identical uncertainty. The uncertainties of the detected control points are mainly determined by camera pose, the slant of the chessboard and the inconsistent imaging capabilities of the camera. The negative influence of the uncertainties that are induced by the two former factors can be eliminated by adequate data sampling. However, the last factor leads to the detected control points from some sensor areas having larger uncertainties than those from other sensor areas. This causes the final calibrated parameters to overfit the control points that are located at the poorer sensor areas. In this paper, we present a method for measuring the uncertainties of the detected control points and incorporating these measured uncertainties into the optimization model of the geometric calibration. The new model suppresses the influence from the control points with large uncertainties while amplifying the contributions from points with small uncertainties for the final convergence. We demonstrate the usability of the proposed method by first using eight cameras to collect a calibration dataset and then comparing our method to other recent works and the calibration module in OpenCV using that dataset.

## 1. Introduction

The geometric calibration of cameras has been studied extensively for imaging systems in computer vision [1]. Widely used tools for camera calibration include the standard MATLAB toolbox and OpenCV routines [2]. In general, the imaging capabilities of cameras are similar across the imaging field and the non-linear optimization solvers of most existing works have been based on this assumption of uniform imaging capabilities. However, when an imaging system incorporates a lens or sensor with an off-axis or field curvature that was induced by a manufacturing deviation or physical collision, the imaging capabilities become different at the different positions of the camera sensor.

Traditional calibration methods tend to lack accuracy for systems with inconsistent imaging capabilities. The uncertainties of the detected control points that are located in the poor areas of the sensor are larger than those in good areas of the sensor, which causes the calibration result to overfit the control points that are located in the poor sensor areas. In the calibration process, camera pose and the slant of the chessboard also introduce uncertainties in the detected control points. However, the negative influence of the uncertainties that are induced by these two factors can be relaxed or eliminated through adequate data sampling, which balances the contributions from different sensor areas in the final convergence of the optimization. By contrast, the uncertainties of the control points that are induced by the inconsistent imaging capabilities of an imaging system cannot be tackled with data sampling.

**Figure 1:**
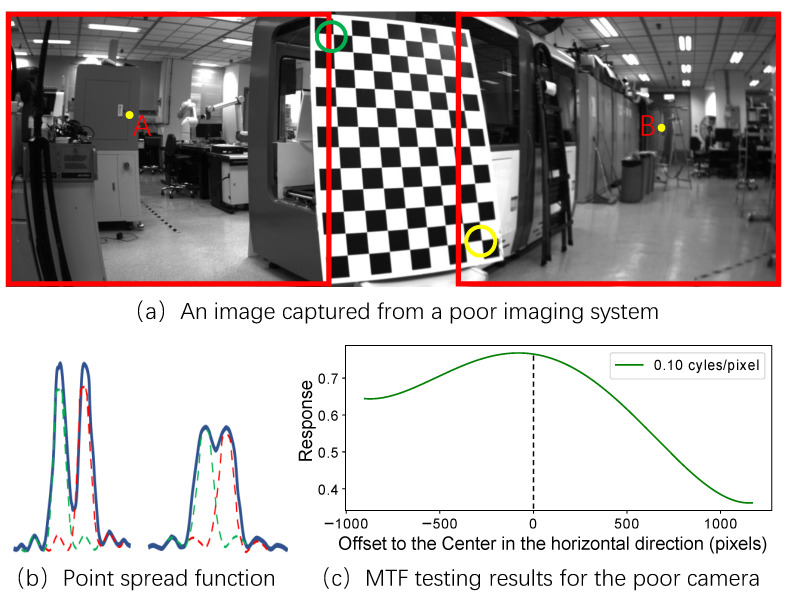
(**a**) shows a cropped image and the corresponding full image was captured by a camera with inconsistent imaging capabilities. (**c**) illustrates the MTF testing results for the camera. The right-hand part of the image is more blurred than the left-hand part and the control point in the yellow circle is more blurred than that in the green circle. (**b**) illustrates the two point spread functions for the two adjacent pixels at positions A and B in the top image. The blue curves in (**b**) depict the superposition of the point spread functions of the two adjacent pixels. The response in (**c**) indicates the spatial frequency response (SFR).

Figure 1a shows an image that was captured from a camera with inconsistent imaging capabilities. The left areas in this image are prominently sharper than the right areas. Figure 1b illustrates the two point spread functions for the two adjacent pixels at positions A and B in the image. The blue curves in Figure 1b depict the superposition of the point spread functions of the two adjacent pixels. Figure 1c shows the modulation transfer function (MTF) testing results from the horizontal pixels of the imaging system at an indicate spatial frequency. MTF testing results express the imaging capability of a camera. Utilizing traditional calibration methods to calibrate this camera would cause the calibrated parameters to overfit the control points located in the right-hand area of the camera sensor. This would cause the calibration result not to be globally optimal and to lack accuracy in other sensor areas.

The method that was introduced in [3] tries to estimate the uncertainties of the control points and utilize them to balance the contributions of different control points in the final convergence. This method takes the autocorrelation matrices of the detected control points as an estimation of the inverse covariance matrices of the control points. However, the relationship between the autocorrelation matrices and the standard deviation of the control points is a multi-mapping function, which causes the estimated uncertainties to be disproportional to the real standard deviations of the control points. With incorrect uncertainties, this method cannot handle the problem of inconsistent imaging capabilities.

In this paper, we present a geometric calibration method for dealing with the problem for which traditional methods tend to lack accuracy when imaging systems have inconsistent imaging capabilities. We designed a novel method to measure the uncertainties of the detected control points. Our measured uncertainties were single-mapped to the standard deviation of the control points. Then, we incorporated the measured uncertainties into the optimization process of camera calibration. This new optimization suppressed the contributions from the points with large uncertainties. To demonstrate the effectiveness of our method, we built a calibration dataset using eight different imaging systems, which consisted of a very poor lens, three poor lenses and four good lenses. We compared our method to those in [3,4] and to the calibration module of OpenCV using that dataset. In summary, the contributions of our work are as follows:

- We proposed a method to estimate the inverse covariance matrix of detected control points;
- We incorporated the measured uncertainties into the optimization process of camera calibration and proved the effectiveness of our method in eight imaging systems;
- We measured the MTF of the imaging systems and analyzed the MTF results for the poor imaging systems.

## 2. Related Works

Related articles can be divided into three categories: calibration with special objects, calibration without calibration targets and learning-based calibration works.

**With calibration targets.** Flexible camera calibration [5] is a pioneering approach that presents a pipeline to calibrate cameras from a printed chessboard. Some studies [6,7,8,9] have explored different calibration targets, such as lines, square patterns, circles, ring patterns and special markers, while others [10,11,12,13,14] have focused on the distortion models of lenses and have presented better calibration accuracies by improving previous lens distortion models. Some studies [15,16] have promoted calibration performance through extracting high-quality target points, others [17,18,19,20] have extended the standard camera calibration pipeline to some degenerating cases and one study [4] proposed an iterative refinement method to improve the localization accuracy of the control points. Although these works have analyzed the camera calibration process from many perspectives, they have not dealt with imaging systems with inconsistent imaging capabilities.

**Without calibration targets.** Geometric-based calibration without calibration targets is also a popular research area. Some studies [21,22,23,24] have extracted geometric cues from images, such as vanishing points, lines, coplanar circles and repeated texture patterns, in order to recover camera parameters. Among them, ref. [21] only utilized one image of two coplanar circles with arbitrary radii to calibrate the camera. Following the idea of [21], Colombo et al. proposed an approach [22] for camera calibration using an image of at least two circles arranged coaxially, while [23] presented an automatic approach to camera calibrate by straightening the slanted man-made structures in the input image. Furthermore, ref. [24] relaxed the requirement of scenes and detected repeated coplanar patterns to perform camera calibration. These methods can be used with images taken outside of the lab and present good calibration accuracy in environments with Manhattan buildings. However, since geometric-based methods rely on detecting and processing specific cues, such as straight lines, they lack robustness for images taken in unstructured environments, with low-quality equipment or under difficult illumination conditions.

**Learning-based calibration.** Some of the most recent works [25,26,27,28] have leveraged the success of convolutional neural networks and have presented learning-based methods to estimate camera parameters. Some studies [27,28] have been able to estimate camera parameters from a single image and have reported a good calibration accuracy. Compared to geometric-based calibration methods, learning-based approaches relax the requirements of the Manhattan environments. For example, ref. [27] estimated camera parameters from forest images. Although these learning-based methods have obtained excellent results, they are a black box and we cannot directly analyze internal details using these methods.

In this paper, we propose a new method to calibrate cameras with inconsistent imaging capabilities. Our method measures the uncertainties of detected control points and incorporates those measured uncertainties into the estimation model. In contrast to the measured uncertainties in [3], our uncertainties are single-mapped to the standard deviations of the control points.

## 3. Preliminary

In this section, we first introduce the notation that is used in this paper. Then, we briefly introduce the point detection of the chessboard pattern and the canonical camera calibration model.

### 3.1. Notations

Let $C=\{c_{i}^{j}=\left(u_{i}^{j},v_{i}^{j}\right)\;|\;i=1\cdots N,j=1\cdots M\}$ denote the set of detected control points, where *N* represents the number of control points on the calibration pattern and *M* indicates the number of images being used for the camera calibration. $P_{i}=\left(x_{i},y_{i},0\right)$ denotes the space coordinates of the ith point on the calibration board, $T=\{T_{j}|j=1\cdots M\}$ represents the observation poses of the *M* images and θ indicates the intrinsic parameters that are to be calibrated.

### 3.2. Control Point Detection

We adopted the chessboard as our calibration target, which is one of the most prevalent calibration objects. The points of the chessboard were detected using the Harris interest point detector [15] and then refined using an iterative sub-pixel localization with a gradient-based search. The search equation was defined as:

(1) $$\begin{array}{c} \sum_{p\in N_{c}}\nabla I\left(p\right)\cdot \left(c-p\right)=0, \end{array}$$

where $N_{c}$ indicates the set of pixels located within the neighborhood of control point c, the operation · signifies a matrix multiplication operation and ∇I(p) denotes the image gradient at location p. The gradient-based search problem could be solved by:

(2) $$\begin{array}{c} c={\left(\sum_{p\in N_{c}}\nabla {I(p)}^{T}\cdot \nabla I(p)\right)}^{-1}\sum_{p\in N_{c}}\nabla {I(p)}^{T}\cdot \nabla I(p)\cdot p, \end{array}$$

where ${\left(\cdot \right)}^{-1}$ denotes the inverse operation of the matrix.

### 3.3. Canonical Camera Calibration

The calibration process comprised two stages: calculating the initial calibration parameters with a closed-form solution and refining the estimated parameters with a non-linear optimization solver. This came down to the minimization of the reprojection error:

(3) $$\begin{array}{c} \underset{T_{j},\theta }{\arg\min}\sum_{i,j}{∥\pi \left(P_{i},T_{j},\theta \right)-c_{i}^{j}∥}_{2}^{2}, \end{array}$$

where the projection function π(·) projects a space point $P_{i}$ to the image plane and ${∥\cdot ∥}_{2}$ indicates L2 normalization. The perfect convergence of Equation (3) requires the detected control points to have an identical uncertainty.

There are three main factors that determine the uncertainties of the detected control points: the inconsistent imaging capabilities in different sensor areas, the different observation poses and the diverse slants of the chessboard. These three factors usually cause the detected control points to have non-isotropic Gaussian distributions and different uncertainties. However, since the latter two factors are related to data sampling, the negative influence of the different uncertainties of the detected control points can be relaxed or eliminated through the use of proper data sampling. With proper calibration data, the different sensor areas make similar contributions to the final convergence of Equation (3). By contrast, the first factor, the inconsistent imaging capabilities of an imaging system, causes the uncertainties of the control points in “poor” sensor areas to be larger than those in “good” sensor areas and this cannot be tackled with data sampling. This discrepancy causes the estimated parameters to overfit the areas with larger uncertainties.

Thus, we propose a method to measure the uncertainties of the detected control points and involve those measured uncertainties in the final optimization process.

## 4. Approach

To deal with the inconsistent imaging capabilities in different sensor areas, we incorporated the uncertainties of the detected points into the optimization stage. This amplified the contributions of the control points with small uncertainties and decreased the contributions of the control points with large uncertainties. Following the definition in the previous section, the new optimization model was:

(4) $$\begin{array}{c} \underset{T_{j},\theta }{\arg\min}\sum_{i,j}\pi {\left(P_{i},T_{j},\theta \right)}^{T}\;{\Sigma_{i}^{j}}^{-1}\pi \left(P_{i},T_{j},\theta \right), \end{array}$$

where $\Sigma_{i}^{j}$ is a 2 × 2 matrix and represents the uncertainties of the detected control point $c_{i}^{j}$. Equation (4) can be solved by Gauss–Newton or Levenberg–Marquardt optimizers [29]. To retain the effectiveness of the optimization process, the measured uncertainties have to be proportional to the real variance of the detected point coordinates. Now, we explain how to measure the uncertainties of the detected control points.

### 4.1. Uncertainty Measurement for Detected Control Points

For the chessboard pattern, the influence of the observation pose and the slant of the board on the control points is entirely represented by the open angle φ and rotation angle ϑ of the detected control point. Moreover, a poor imaging capability leads to blurred imaging results. Thus, in our inconsistent imaging system, some image areas were clear and others were blurred. The general strategy to measure the uncertainties of the positions of the detected control points is to compute the autocorrelation matrix C for a window of a given size around the point [3,15]. Concretely, this strategy considers the autocorrelation matrix C to be an estimation of the inverse of the covariance matrix for the control point, which is a partially correct conclusion. When we fixed the data sampling conditions, such as observation pose, signal-to-noise ratio (SNRs) and blur levels, a larger autocorrelation matrix corresponded to a smaller covariance matrix. However, the above conclusion cannot hold for two arbitrary control points; specifically, it is a multi-mapping relationship.

**Figure 2:**
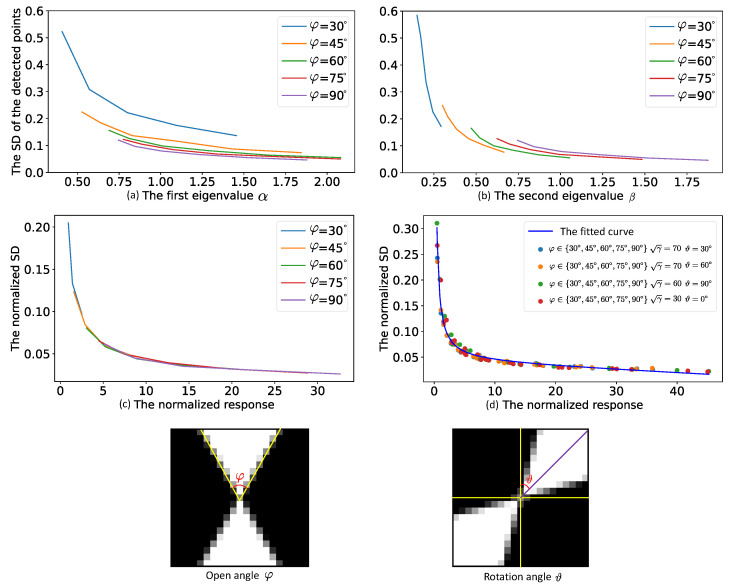
(**a**,**b**) illustrate the changes in the standard deviations of the control points with regard to the increase in the eigenvalues of the autocorrelation matrix of the control points at five open angles. The same eigenvalue corresponds to different standard deviations at different open angles. The two points at the bottom of the figure represent the open angle φ and the rotation angle ϑ. (**c**) shows the changes in the normalized standard deviations of the control points with regard to the increase in the normalized point response at five different open angles. (**d**) illustrates the fitted curve for the relationship between the normalized responses and the normalized standard deviations at different open angles (φ), rotation angles (ϑ) and SNRs (γ).

We let (α,β) and $\left(v_{\alpha },v_{\beta }\right)$ denote the eigenvalues and eigenvectors of the autocorrelation matrix C, respectively. Figure 2a,b illustrate the relationship between the eigenvalues (α,β) and the standard deviations (SDs) of the detected control points at different open angles. The SD illustrated in Figure 2 is a statistical value and was calculated using four steps: (1) we generated a large number of point maps by providing different open angles, SNRs, blur levels and rotation angles; (2) we detected the control points and refined them on the point maps; (3) we collected a subset that satisfied the indicating conditions; and (4) finally, we gathered the SDs of the detected control points in the subset for different eigenvalues. We could see that the eigenvalues (α,β) could not uniquely determine the real variance of a point.

We dealt with this multi-mapping problem by incorporating a self-defined SNR γ and two normalizing operations. The SNR γ was defined as:

(5) $$\begin{array}{c} \gamma =\frac{{\left(white-black\right)}^{2}}{\sigma^{2}}, \end{array}$$

where white and black are the mean values of the white blocks and black blocks around the detected point, respectively. Specifically, we analyzed the intensity histogram of the local area and automatically selected two thresholds to detect the white and black blocks. $\sigma^{2}$ was the variance of the image-capturing noise in the intensity domain and could be pre-calibrated using [30]. We let $x=\frac{\alpha \beta }{\gamma^{2}\sigma^{4}}$ indicate the normalized point response and $y=100\sqrt{\gamma }\mathrm{SD}$ represent the normalized SD. The relationship between the normalized response and the normalized SD is shown in Figure 2c,d. We could see that the relationship was stable with regard to the changes in the open angles, SNRs, rotation angles and blur levels. Therefore, we could build a function to predict the normalized SD from a normalized point response. The prediction function was:

(6) $$\begin{array}{c} f\left(x\right)=y=a\frac{1}{x}+bx+c, \end{array}$$

where *a*, *b* and *c* are the fitting parameters and the fitted curve is shown in Figure 2d.

We estimated the inverse covariance matrix $\Sigma^{-1}$ of the control point by:

(7) $$\begin{array}{c} \Sigma^{-1}=\left[v_{\alpha },v_{\beta }\right]\cdot \left[\begin{array}{cc} \frac{\alpha +\beta }{\beta }{\left(\frac{100\sqrt{\gamma }}{f(\frac{\alpha \beta }{\gamma^{2}\sigma^{4}})}\right)}^{2}\; & 0\\ 0 & \frac{\alpha +\beta }{\alpha }{\left(\frac{100\sqrt{\gamma }}{f(\frac{\alpha \beta }{\gamma^{2}\sigma^{4}})}\right)}^{2} \end{array}\right]\cdot {\left[v_{\alpha },v_{\beta }\right]}^{T}. \end{array}$$

The eigenvectors $\left(v_{\alpha },v_{\beta }\right)$ in the above equation decoupled the estimated SD to the horizontal and vertical directions of the image. The coefficients $\frac{\alpha +\beta }{\beta }$ and $\frac{\alpha +\beta }{\alpha }$ in Equation (7) ensured the estimated inverse covariance matrix had a similar uncertainty to the predicted SD. Since **inconsistent imaging capabilities** cause image coordinate-related blurring, we focused on how the SDs of the detected control points changed for different open angles, SNRs and rotation angles when we changed the blur levels of the points.

**Point standard deviation vs. open angle.** The open angles of the detected points of the chessboard pattern mainly represent the observation pose and the slant of the board. We fixed the SNR and rotation angle and only changed the blur levels of the point maps at different open angles. Then, we gathered the SDs of the detected control points. Figure 2c illustrates the relationship between the normalized point responses and the normalized SDs. Although the calculated normalized responses for different open angles were distributed in different ranges, the change in the normalized SDs with respect to the normalized responses showed excellent consistency throughout the whole curve.

**Figure 3:**
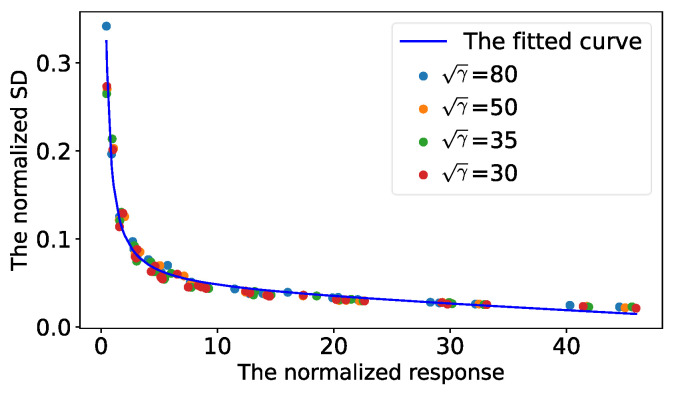
An illustration of the normalized SDs vs. the change in SNRs (γ). The blue, orange, green and red points denote the sampling from the SNRs $80^{2}$, $50^{2}$, $35^{2}$ and $30^{2}$, respectively. The blue curve is fitted by all sampling. It can be seen that the fitted curve successfully describes the relationship between the normalized SDs and the normalized responses at different SNRs.

**Point standard deviation vs. SNR.** SNR is another key component for analyzing the SDs of the detected control points. Due to the inevitable lens shading problem, captured images are usually brighter (larger signal) in the central areas and darker (smaller signal) in the border areas. Moreover, the image capturing process involves captured noise and the noise variance can be calibrated by the method presented in [30]. In short, the SNRs of detected control points are related to the point positions, exposure times, the aperture size of the camera and the environmental illumination. The SNR could be formulated using the definition in Equation (5). Figure 3 illustrates the influence of SNR on the SDs of the detected control points. The relationship between the normalized point responses and the normalized SDs were stable for the different SNRs.

**Figure 4:**
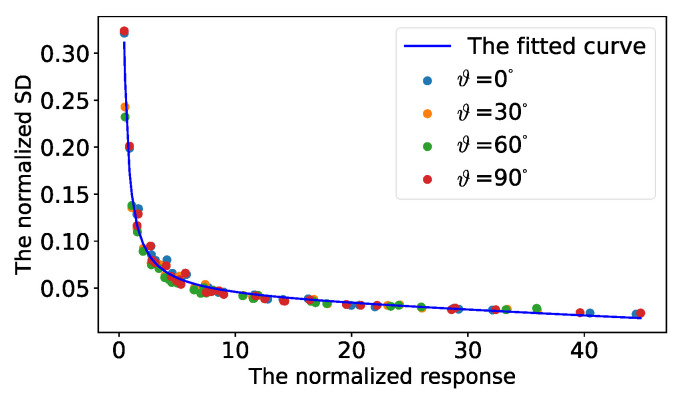
An illustration of the normalized SDs vs. the change in rotation angles. The blue, orange, green and red points denote the sampling from the rotation angles 0, 30, 60 and 90 degrees, respectively. The blue curve is fitted by all sampling. It can be seen that the fitted curve successfully describes the relationship between the normalized SDs and the normalized responses at different rotation angles.

**Point standard deviation vs. rotation angle.**Figure 4 illustrates the influence of the rotation angle on the SDs of the detected control points. Due to the natural rotation invariance of eigenvalue decomposition, the normalized responses showed a good consistency with the change in rotation angle.

In summary, we transferred the multi-mapping function (the eigenvalues of the detected points with regard to the SDs of the detected points) to a single-mapping function (the normalized point responses with regard to the normalized SDs of the control points). Benefiting from the single-mapping function, we could predict the control point SD using Equation (6), estimate the inverse covariance matrix using Equation (7) and also incorporate the estimated matrix into the optimization model defined in Equation (4).

### 4.2. Point Standard Deviation vs. Kernel Size

**Figure 5:**
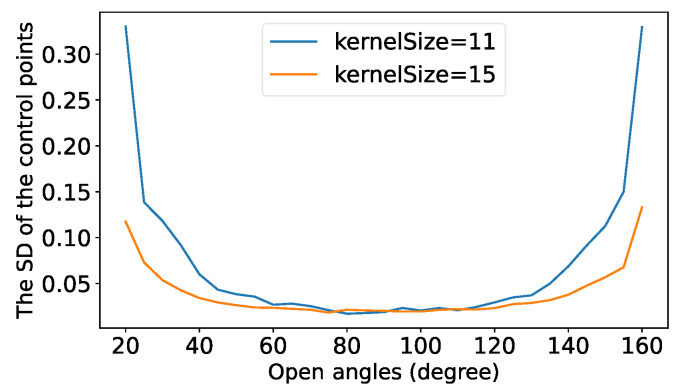
An illustration of the change in the standard deviations of the detected control points with respect to the open angles of the control points at two different kernel sizes.

The kernel size determines how many pixels are used to refine the positions of the detected control points and thus, determines the size of $N_{c}$ in Equation (2). We found that increasing the kernel size could increase the detection stability for the control points of small or large open angles. Figure 5 illustrates the SDs of the detected control points with respect to the increase in open angel at different kernel sizes. In our implementation, we increased the kernel size of a detected point when its open angle was located in the unstable range and the predicted SD of the point was large. The details of the switching of the kernel sizes are described in Section 5.4.

### 4.3. Iterative Refinement

Similar to the method presented in [4], we also iteratively localized the control points, which could reduce the interference of lens distortion.

**Figure 6:**
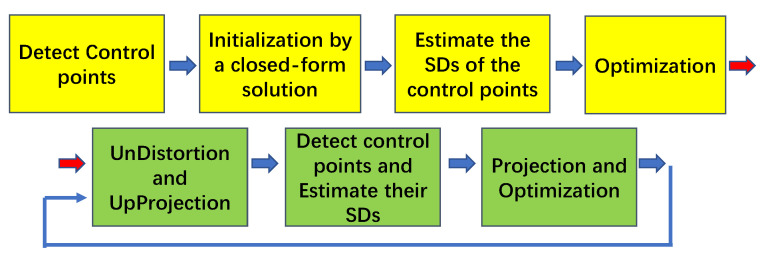
An illustration of the pipeline of our method. The four yellow blocks indicate the standard parts of the calibration process and the three green blocks denote the iterative parts of the calibration process.

The pipeline is illustrated in Figure 6. The yellow blocks indicate the standard parts of the calibration process and the green blocks denote the iterative parts of the calibration process. The up-projection stage projects the undistorted image onto the fronto-parallel plane through homography transformation. The transformation matrix is composed of the estimated camera poses and the intrinsic parameters. The reprojection stage utilizes the estimated parameters to project the detected control points onto the original distorted image plane. The influence of the deviation that is contained in the estimated parameters is almost completely offset in the process of up-projection and reprojection.

## 5. Experiments

**Figure 7:**
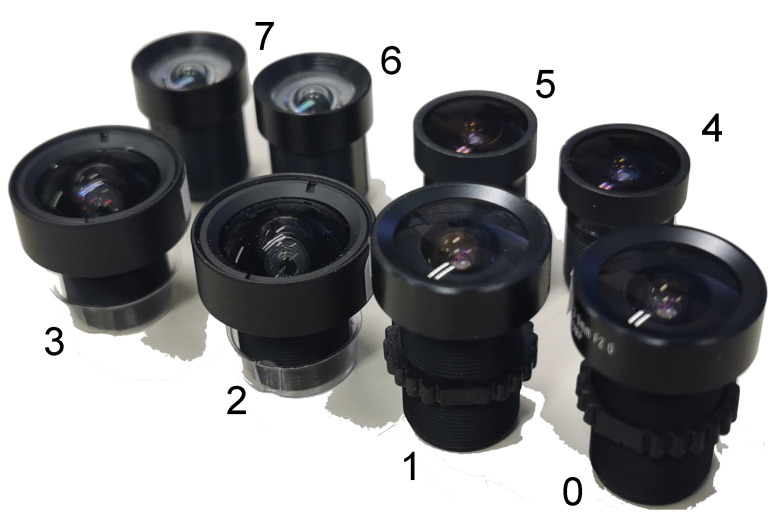
An illustration of the eight lenses that were used in our experiments. Lenses 0 to 3 are high resolution, while Lenses 4 to 7 are low resolution. The imaging capability of Lens 0 is very inconsistent, while Lens 1, Lens 4 and Lens 5 are a little bit inconsistent and the remaining four lenses are good.

We evaluated our method using a real dataset. The real dataset was collected from a setup composed of eight lenses (as shown in Figure 7) and six sensors. They formed four high-resolution imaging systems and four low-resolution imaging systems. The resolutions of the former were up to 3088×2076 and they contained two uEye UI-3881LE sensors from IDS, two Lensagon BK5M3920 lenses from Lensation and two Pixoel S03428-5M lenses from Pixoel. The resolutions of the latter were 1280×1024 and they included four CM3-U3-13Y3C cameras from Point Grey Research and four low-resolution lenses.

### 5.1. Consistency Analysis for Imaging Systems

Before evaluating the performance of our method, we analyzed the consistency of the imaging capabilities of our imaging systems using MTF testing. The MTF result at a pixel coordinate indicates how much information can be retained for the input with different spatial frequencies at this position. In short, a small MTF result corresponds to a blurred image. To measure the MTF, we used the slanted-edge gradient analysis method [31], which includes the following steps: (1) taking images of the ISO 12233 test chart, which contains black slanted edges and curved bars on a white background; (2) manually labeling the regions of interest (ROIs); (3) finding the slanted edges in every ROI and computing the MTF array for every row of the ROIs; (4) applying the discrete Fourier transform (DFT) to the calculated MTF array to generate an MTF curve over the spatial frequency for every ROI.

**Figure 8:**
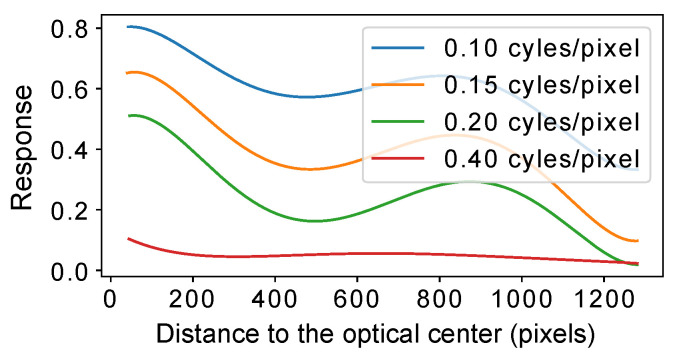
This figure shows the MTF testing results for Lens 0 (Lensagon). A large cycle or pixel number indicates a large spatial frequency. It can be seen that the responses for an identical frequency were diverse in different sensor areas. The responses of the central pixels were higher than those of the border pixels.

**Figure 9:**
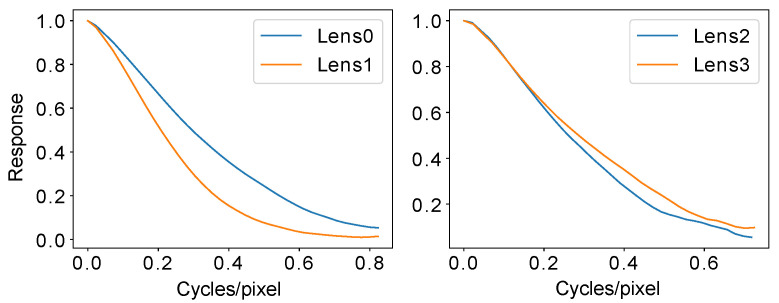
These two plots illustrate the MTF testing results at the same pixel positions for a sensor with two Lensagon lenses and two Pixoel lenses. Although the two lenses in each plot were the same model, their imaging capabilities were diverse.

Figure 8 illustrates the MTF results of a high-resolution imaging system. The figure shows the changes in spatial frequency responses (SFRs) with the increasing pixel distances to the optical center. It can be seen that the imaging capability of the lens is inconsistent, with the capability of central areas being better than that of the border areas. Meanwhile, Figure 1c shows an imaging system that has a higher imaging capability on its left than on its right. Figure 9 illustrates the testing results at the same image position for two Lensengan BK5M3920 lenses and two Pixoel S03428-5M lenses. It can be seen that there was a difference, even though they are the same model.

In summary, the MTF test results proved that some imaging systems had inconsistent imaging performance in different sensor areas.

### 5.2. Generation of Training and Testing Datasets

We built a calibration dataset using the eight imaging systems to collect calibration data. From a sequence of *N* images collected from an imaging system, we randomly selected *M* images as a training sequence and took the remaining N-M images as the corresponding testing sequence. We generated 50 training and testing sequences for each imaging system; thus, we produced 400 training and testing sequences in total.

### 5.3. Evaluation of Our Method

To demonstrate the effectiveness of our method, we compared it to those in [3,4], as well as to the calibration module of OpenCV. The method in [4] is an iterative point refinement approach, the calibration pipeline of which requires repeatedly implementing the optimization process. Meanwhile, the approach in [3] takes the autocorrelation matrices of the detected control points as an estimation of the inverse covariance matrices of the control points and incorporates the estimated inverse covariance matrices into the optimization stage.

The metric that we used to evaluate the calibration results was the average projection error. The average projection error $e_{proj}$ for the *j*th image was defined as:

(8) $$\begin{array}{c} e_{proj}\left(j\right)=\frac{1}{N}\sum_{i=1}^{N}{∥\pi \left(P_{i},T_{j},\theta \right)-c_{i}^{j}∥}_{2}^{2}, \end{array}$$

where *N* represents the number of control points in the calibration pattern, $c_{i}^{j}$ denotes the detected control points and $P_{i}$ is the space coordinate of the ith point on the calibration board. Furthermore, $T_{j}$ represents the observation pose of the *j*th image and θ indicates the estimated intrinsic parameters. In short, considering the estimated intrinsics θ, the average projection error $e_{proj}$ for the *j*th image was calculated using two steps: (1) estimating the pose $T_{j}$ by solving a standard PnP [32] and (2) computing the average projection error of all of the detected points in the image based on the intrinsic θ and the estimated pose $T_{j}$.

**Figure 10:**
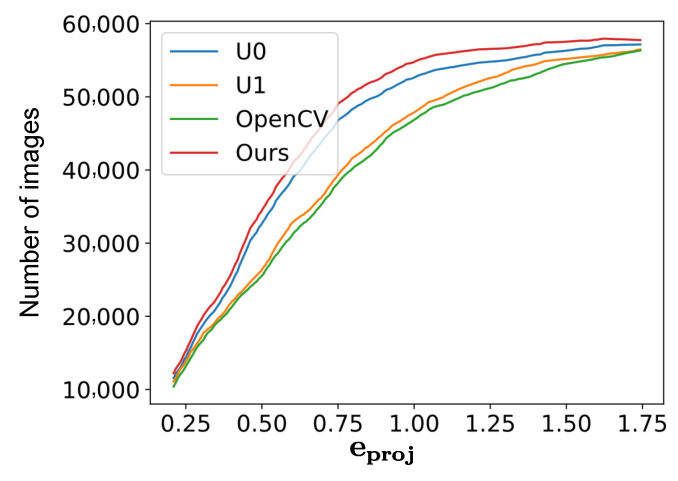
The value on the vertical axis indicates the accumulated number of images whose average projection errors were smaller than $e_{proj}$. A larger number indicates a better performance.

To avoid redundant references, henceforth we use U0, U1 and OpenCV to indicate the solutions from [3,4] and the OpenCV calibration module, respectively, while Ours indicates the results from our method. Among the competing methods, U0 is an iterative calibration method and it iteratively refines the control points. U1 applies a naive method to measure the uncertainties of the points and involves the measured uncertainties in its calibration process. Figure 10 illustrates the statistical performance of these four methods on images collected from all camera systems. The values on the vertical axis are the accumulated number of images whose average projection errors were smaller than $e_{proj}$. A larger number denotes a better calibration performance. The performances of U0 and U1 were a little better than that of OpenCV, while our method presented the best accuracy out of the four methods. 

In Table 1, we used the overall average reprojection error $e_{proj}^{all}$ as a metric and compared the accuracies of the four methods using our eight imaging systems. The metric $e_{proj}^{all}$ for the *k*th camera system was calculated by:

(9) $$\begin{array}{c} e_{proj}^{all}\left(k\right)=\frac{1}{M_{k}}\sum_{i=1}^{M_{k}}e_{proj}\left(i\right), \end{array}$$

where $e_{proj}$ is defined in Equation (8) and $M_{k}$ indicates the number of images collected by the *k*th camera system. “High” in the second column of the table shows that the row corresponds to a high-resolution imaging system and “Low” represents a low-resolution imaging system. “Large” and “Small” in the third column represent that the amplitude of the lens distortion of the imaging system was large or small, respectively. The fourth column describes whether the imaging capabilities of the system were consistent. The performance of the iterative method U0 was better than that of OpenCV when the amplitudes of the imaging system lens distortions were large. This was because the distorted point map interfered with the detection of the control points. Since the relationship between the autocorrelation matrices used in U1 and the inverse covariance matrices of the control points are a multi-mapping function, the accuracies of U1 were unstable and were sometimes worse than those of OpenCV. Our method inherited the advantages of U0 and the estimated covariance matrices used in our method were almost proportional to the real covariance matrices of the control points. Therefore, our method presented a more robust performance than the other three methods. It can be seen that our results showed obvious improvements in imaging systems with inconsistent imaging capabilities.

### 5.4. Switching Kernel Size during Point Detection

**Figure 11:**
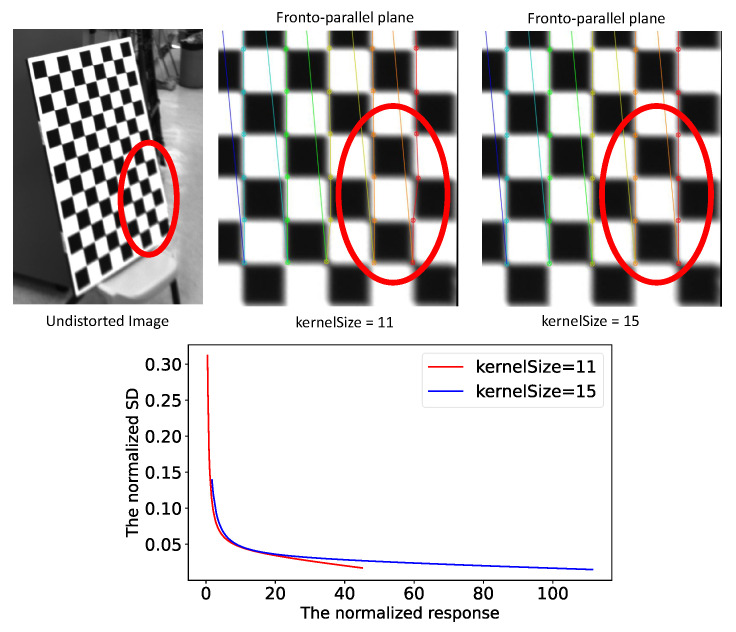
The left-hand image shows the cropped chessboard from an undistorted image. The middle and right-hand images are the fronto-parallel plane versions of the cropped chessboard. The red circles indicate the same image area. The detected control points in the fronto-parallel image showed obvious deviations when the kernel size was 11. The bottom graph illustrates the relationship between the normalized point responses and the normalized standard deviations of the detected control points.

We found that increasing the kernel size could improve the stability of the detected control points for points with small or large open angles. We calculated the open angle of a control point $c_{i}$ by:

(10) $$\begin{array}{c} \mathrm{angle}=\arccos\frac{{\left(c_{i+1}-c_{i}\right)}^{T}\cdot \left(c_{i+row}-c_{i}\right)}{{∥c_{i+1}-c_{i}∥}_{2}\cdot {∥c_{i+row}-c_{i}∥}_{2}}, \end{array}$$

where row indicates the number of control points in a row of the chessboard. An example for switching kernel size is shown in Figure 11. To visualize the detected control points, we reprojected the undistorted image onto the standard fronto-parallel plane. The detected control points located in the red circle in the figure showed deviation when the kernel size that was used in the point detection stage was 11, while the accuracy of detected control points was obviously improved when we switched the kernel size to 15. Moreover, the relationship between the normalized point responses and the normalized SDs of the control points was different for different kernel sizes. The difference is shown in the bottom graph of Figure 11. When we increased the kernel size in the refinement of the control points, we were required to utilize different fitted parameters to predict the normalized SDs of the control points.

### 5.5. Calibration for Stereo

We could transfer our method to stereo calibration easily. Similar to the monocular case, we first estimated the inverse covariance matrices for all control points that were detected in the image pairs and then incorporated the computed inverse covariance matrices for the projection residuals of the control points in left-hand and right-hand views. As all points from the image pairs were independent of each other, we did not require any additional modifications.

### 5.6. Notice

A high-accuracy image noise calibration is essential for our method. The predicted standard deviations of the control points were shifted with the calibrated variance of the image noise. Therefore, an inaccurate image noise calibration result would cause the predicted uncertainties to tend to have different scales across various pixel positions and different orientations of the target, which would make the uncertainties of those pixels incomparable.

### 5.7. Runtime Analysis

Since the refinement of the points is inevitable in the general geometric calibration pipeline, the autocorrelation matrix C could be obtained without additional time costs. Our method requires the implementation of eigenvalue decomposition for the autocorrelation matrix C of all control points. The decomposition of a point took about 0.01 ms in a Linux system with an Intel Core i7-8700 CPU with 3.20 GHz. Moreover, we utilized the neighboring points of these control points to calculate their open angles and thus, there was no additional time cost.

## 6. Conclusions

In this paper, we presented a geometric calibration method to deal with the problem for which traditional calibration methods tend to lack accuracy when imaging systems have inconsistent imaging capabilities. We demonstrated the superiority of the proposed method by first using eight cameras to collect a large calibration dataset and then comparing the proposed method to two recent works [3,4] and the OpenCV calibration module. For cameras with inconsistent imaging capabilities, the proposed method achieved better calibration accuracies than those of competing methods. Meanwhile, for cameras with consistent imaging capabilities, the calibration accuracies of the proposed approach were similar to those of the competing methods. The proposed method adopted a novel approach, which involved the SNRs of the control points and two new normalizing operations to measure the uncertainties of the detected control points in chessboard images. The new method ensured that the calculated uncertainties of diverse control points were comparable across various pixel positions and different orientations of the target. Benefiting from the comparable uncertainties, the proposed method increased the consideration of the constraints of those control points with low uncertainties, which allowed the method to achieve a better calibration performance for poor imaging systems. In the experimental section, we utilized the MTF tool to analyze the imaging capacities of the eight cameras. The quantitative calibration results were also discussed for these eight camera systems. In future work, we plan to investigate the negative influence of motion blur in the geometric calibration pipeline and explore how to obtain a good calibration accuracy from an image sequence with large motion blur.

## Figures and Tables

**Table 1:** Calibration accuracy.

Id	Resolution	Distortion	Consistency	OpenCV	U0	U1	Ours
1	High	Large	Poor	1.173	0.935	0.906	0.815
2	High	Large	Poor	0.941	0.796	0.925	0.764
3	High	Small	Good	0.359	0.358	0.394	0.356
4	High	Small	Good	0.204	0.211	0.237	0.205
5	Low	Large	Poor	0.857	0.705	0.862	0.689
6	Low	Large	Poor	0.936	0.832	0.871	0.713
7	Low	Large	Good	0.301	0.279	0.323	0.278
8	Low	Large	Good	0.485	0.431	0.517	0.442

## Data Availability

Not applicable.
